# The complete mitochondrial genome of a satin moth: *Leucoma chrysoscela* (Lepidoptera: Erebidae, Lymantriinae)

**DOI:** 10.1080/23802359.2022.2055977

**Published:** 2022-03-30

**Authors:** Jing Li, Qing Lv, Ai-bing Zhang

**Affiliations:** College of Life Sciences, Capital Normal University, Beijing, P. R. China

**Keywords:** *Leucoma chrysoscela*, mitochondrial genome, Erebidae, Lymantriinae, phylogenetic analysis

## Abstract

We describe the mitogenome sequence of *Leucoma chrysoscela* (Collenette, 1934) collected in the Longtan National Forest Park, which is located in the southeast of China. The assembled mitogenome is 15,508 bp in length and consists 13 protein coding genes, 22 transfer-RNA genes, 2 ribosomal-RNA genes, and one A + T rich region. The most common start codon for 13 PCGs is ATT and the most common termination codon is TAA. The overall G + C content was only 20.45% in the heavy strand. The result of phylogenetic analysis shows that the relationship of *L. chrysoscela* is close to the species in the same subfamily Lymantriinae.

The satin moth, *Leucoma chrysoscela* (Lepidoptera: Erebidae, Lymantriinae) is a serious defoliator of willow and tea leaves which might cause severe damage in forests (Holloway 1999). Most previous studies on genus *Leucoma* have focused on *L. salicis*, which is a worldwide pest not only influencing quality and quantity of tea products but damaging roadside and garden trees in urban areas as well (Chao 2003; Jakubowska et al. 2005). The causes of outbreak of *L. salicis* have aroused wide attention, but it was not completely clear yet and may be related to the host plants, climatic conditions, parasitoids and epizootic factors (Wagner and Leonard 1979, 1980; Ziemnicka 2008). The *L. chrysoscela* and *L. salicis* were allopatric species in China, *L. chrysoscela* generally located in the southeast of China and *L. salicis* mainly found in the northwest (Chao 2003). Although these two species belong to the same genus, they had relatively distinct morphological and genetic differences (Chao 2003; Wang et al. 2015), which might be associated with the environmental adaptive differences. Unlike detailed studies on *L. salicis,* studies on *L. chrysoscela* were quite limited currently. A complete mitogenome of *L. chrysoscela* might provide systematically-informative information for species identification, phylogenetic analysis and evolutionary studies on *Leucoma* and Lymantriinae.

We collected samples of *L. chrysoscela* in the Longtan National Forest Park, Guangxi Province, China (36°34′3″N and 101°49′17″E) in July 2020. All specimens were preserved in 95% ethanol in the field and stored at 4 °C in the laboratory until DNA extraction. Voucher specimen was deposited at the Entomological Museum of Capital Normal University under the voucher number G200715#Lep. Total genomic DNA was extracted from the leg muscle tissue using the Genomic DNA Extraction Kit (QIAGEN, Hilden, Germany). The purified DNA samples were used to prepare an Illumina TruSeq library and sequenced on the Illumina HiSeq2500 platform. Low quality (ambiguous bases) and short reads (shorter than 95 bp) were filtered during quality control with FastQC v0.11.7 (Andrews 2010), AdapterRemoval v2.2.2 (Schubert et al. 2016) and SOAPec v2.03 (Luo et al. 2012). High-quality reads were assembled and annotated with MitoZ v2.4 (Meng et al. 2019). The DNA sequence was deposited in GenBank under the accession number MW030505. The length of the complete mitochondrial genome of *L. chrysoscela* is 15,508 bp, which contains 37 genes, including 13 protein-coding genes (PCGs), 22 transfer RNA (tRNA) genes, 2 ribosomal RNA (rRNA) genes and 1 control region (A + T-rich region) which is 429 bp in length. For the 13 PCGs, the most common start codon is ATT (*ND2, ND3, ND5, ND6, COX1* and *ATP8*), then is ATG (*ATP6, ND4L, CYTB, COX3* and *ND4*) and ATA (*COX2* and *ND1*); the most common termination codon is TAA (13 protein coding genes except *ND4L* and *ND1*).

The mitochondrial base composition is A 40.24%, T 40.59%, G 7.42%, and C 11.75% in the heavy strand, with an obvious (A + T) % > (G + C) %. Similar situation occurred in the non-coding region in which the (A + T) % was more than 91%. The length of the 22 sequenced tRNA genes range from 64 to 73 bp. The lrRNA is 1311 bp long with an A + T content of 84.29% and the srRNA is 750 bp long with an A + T content of 84.80%.

All 13 protein-coding genes were used to reconstruct the phylogenetic tree with the Maximum Likelihood approach in RAxML 7.9.6 (Stamatakis 2006). Based on the phylogenetic structure, *L. chrysoscela* formed a monophyletic group with *Lymantria umbrosa*, *Lachana alpherakii* and *Gynaephora minora*, which implied *L. chrysoscela* has close relationship with Lymantriinae instead of other subfamilies in Erebidae. The genus *Leucoma* is widely distributed, ranging from the Palearctic to Africa, New Guinea and Australia, and has the highest species diversity in the Oriental tropics (Ziemnicka 2008). Nevertheless, few previous studies have been undertaken on mitogenomes of *Leucoma*, relatively little is known about the phylogenetic relationship of this genus. The complete mitogenome of *L. chrysoscela* offered novel method for species identification and phylogenetic analysis. Further taxon sampling within *Leucoma* and related genera is required to fully resolve the placement of Leucoma. Meanwhile, at the level of superfamilies, the relationships of Noctuoidea were described as (Notodontidae + (Erebidae + (Nolidae + (Noctuidae + Euteliidae) in this study (Figure 1), which was consistent with previous report (Yang et al. 2019). This study will be helpful to understand the phylogenetic relationships of Erebidae and Lymantriinae.

**Figure 1.**
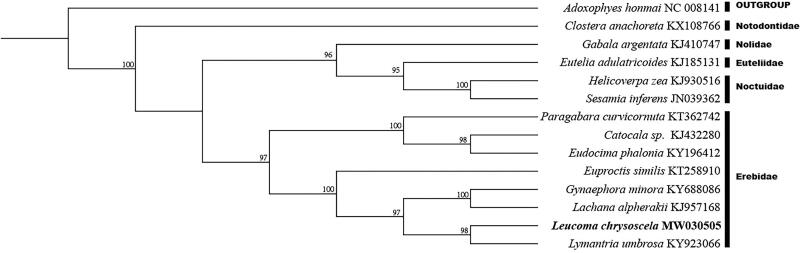
Phylogenetic relationship of 14 species in Lepidoptera based on the concatenated data set of 13 protein-coding genes. Number above each node indicates the ML bootstrap support values. Alphanumeric terms indicate the GenBank accession numbers.

## Data Availability

The data that support the findings of this study are openly available in NCBI at https://www.ncbi.nlm.nih.gov under the accession number MW030505.
